# Upstream Factors Associated With Hospitalization in Black- and Minority-Serving Hospitals

**DOI:** 10.1001/jamanetworkopen.2026.19544

**Published:** 2026-07-17

**Authors:** Louisa W. Holaday, Alina Kung, Yingtong Chen, Karen McKendrick, Bian Liu, Albert Siu

**Affiliations:** 1 Division of General Internal Medicine, Icahn School of Medicine at Mount Sinai, New York, New York; 2 Institute for Health Equity Research, Icahn School of Medicine at Mount Sinai, New York, New York; 3 The Brookdale Department of Geriatrics, Icahn School of Medicine at Mount Sinai, New York, New York; 4 Department of Population Health Science and Policy, Icahn School of Medicine at Mount Sinai, New York, New York; 5 Department of Geriatrics, James J. Peters VA Medical Center, Bronx, New York

## Abstract

**Importance:**

Racial separation of care contributes to racial disparities in health.

**Objective:**

To examine the association of place-based contextual factors with hospitalization in a hospital that disproportionately serves Black patients (Black-serving hospital [BSH]) or a hospital that disproportionately serves racial and ethnic minority patients (minority-serving hospital [MSH]) and whether associations are modified by individual race and ethnicity or whether the hospitalization is elective.

**Design, Setting, and Participants:**

This cross-sectional analysis of data collected from January 2011 through December 2020 from the nationally representative Medicare Current Beneficiary Survey linked with Medicare claims was conducted from March 2023 through September 2025. Participants included all Medicare-linked survey respondents aged 65 years or older who were hospitalized during the study period.

**Exposures:**

Neighborhood disadvantage categorized as 5 groups of the Social Deprivation Index (SDI) and hospital referral region (HRR) residential segregation, with test of effect modification by race and ethnicity and whether the hospitalization was elective.

**Main Outcomes and Measures:**

Hospitalization in a BSH (primary outcome) or MSH (secondary outcome) vs not.

**Results:**

The final sample included 8735 respondents (mean [SD] age, 79.6 [8.2] years; 4967 [56.9%] female; 756 [8.7%] Black, 517 [5.9%] Hispanic, 7093 [81.2%] non-Hispanic White, and 369 [4.2%] multiracial or other). The 8540 with SDI data were evenly distributed in SDI groups 1 through 4 (20.9%-21.4% in each group), with 15.9% in SDI group 5; 77.9% lived in moderately segregated HRRs and 12.1% in highly segregated HRRs. In models fully adjusted for patient-level, HRR-level, and regional covariates, neighborhood disadvantage (adjusted odds ratio [AOR] for most disadvantaged compared with least: 2.03 [95% CI, 1.56-2.63]) and for residential segregation (AOR for high compared with low: 2.99 [95% CI, 1.98-4.52]) were independently associated with hospitalization in a BSH. In stratified models, in the least disadvantaged or segregated areas all patients were unlikely to be hospitalized in a BSH; however, in the most disadvantaged or segregated areas, White patients had a small increase in probability of BSH hospitalization (AOR, 1.70 [95% CI, 1.24-2.34]; *P* = .001; or AOR, 1.98 [95% CI, 1.23-3.18]; *P* = .005, respectively), while Black patients had a larger increase (AOR, 2.77 [95% CI, 1.15-6.60]; *P* = .02; or AOR, 5.55 [95% CI, 1.86-16.57]; *P* = .002, respectively). Elective hospitalizations differed in that race and ethnicity and residential segregation were not independently associated with site of care, neighborhood disadvantage was only marginally significant, and interactions were not significant.

**Conclusions and Relevance:**

This cross-sectional study found an association between site of care and place-based factors, and that it was modifiable by race and ethnicity and whether the hospitalization was elective. Neighborhood disadvantage and residential segregation were independently associated with receiving care in a BSH for Black patients but odds were lower for White patients, and elective hospitalizations had less place- and race-based separation of care. These findings underscore the need for (1) further research to understand these differences, (2) to address place-based contextual factors, and (3) to invest in Black-serving hospitals.

## Key Points

**Question:**

Are place-based contextual factors associated with receiving care in a Black-serving hospital, and are the associations modified by race and ethnicity or whether the hospitalization is elective?

**Findings:**

In this cross-sectional study of data from 8735 respondents to the Medicare Current Beneficiary Survey, neighborhood disadvantage and residential racial segregation were independently associated with hospitalization in a Black-serving hospital. The association differed between Black and White patients, with significantly greater effect sizes for Black patients.

**Meaning:**

These findings suggest that place-based contextual factors are associated with racial disparities in health outcomes through site of care but the association between contextual factors and site of care is modifiable, which warrants further research.

## Teaser

This cross-sectional study investigates the association of neighborhood disadvantage and residential racial segregation with receiving care in a Black-serving hospital (BSH) or minority-serving hospital (MSH) and evaluates potential effect modification by race and ethnicity and elective hospitalization status.

## Introduction

Structural factors contribute to racial disparities in health outcomes, including through differential exposure to neighborhood disadvantage and residential segregation.^1^ Yet racial disparities persist across levels of these place-based contextual factors (henceforth referred to as contextual factors for brevity).^2^ Contextual factors may contribute to health disparities through racial separation of care (ie, patients from different racial groups tend to receive care at different hospitals).^3,4,5^ Hospital facilities were de jure desegregated in 1966 due to Medicare requirements, yet in 2019, half of all hospitalizations among Black Medicare beneficiaries occurred in only 12% of hospitals.^5,6,7,8,9,10^ Hospitals that disproportionately serve Black patients (so-called Black-serving hospitals [BSH]) have lower revenue and profit, lower star ratings, and less access to resource-intensive and specialty care.^11,12,13,14,15,16,17^ Possibly as a consequence of these issues, patients treated in BSH generally have worse outcomes independent of patient and other hospital and health care market characteristics.^4,5,10,18,19,20,21,22,23^ Further supporting that site of care is an important driver of outcomes, within BSH racial differences in outcomes are smaller or not significant.^3,5,21,24,25,26^ Since, by definition, many Black patients receive care in BSH, racial separation of care appears to contribute to racial disparities in health outcomes in the US.

A better understanding of the drivers of racial separation of care may identify intervention points.^27^ Living in a disadvantaged neighborhood is associated with poor health,^28^ which could be due in part to site of care.^3^ Residential racial segregation of hospital referral regions (HRRs) is associated with racial separation of hospital care.^5,9,10,26^ Yet there is evidence that Black patients are more likely to be hospitalized at a BSH even if that hospital is further away.^9^ This evidence suggests the role of additional factors for patient sorting, such as where physicians recommend patients be hospitalized based on race, or patient preference for a BSH,^29,30,31^ which could be suggested by differences between elective and nonelective hospitalizations or differences in the associations by race and ethnicity.

To address these gaps in the literature, we conducted a cross-sectional analysis using data from the Medicare Current Beneficiary Survey (MCBS) linked to Medicare claims. We tested the following hypotheses: (1) that neighborhood deprivation and health care market residential segregation are each associated with hospitalization in a BSH independent of each other, patient characteristics, and other health care market and regional factors; (2) that these associations would differ by race, with a larger effect size for Black compared with White patients; and (3) that associations would differ based on whether the hospitalization was elective. We also examined hospitalizations in a broader group of hospitals that served a disproportionate number of patients from any racial or ethnic minority group (so-called minority-serving hospitals [MSH]).

## Methods

### Sample

We conducted a cross-sectional analysis of MCBS data collected from January 2011 through December 2020. MCBS is a rotating panel survey nationally representative of the Medicare population that oversamples Hispanic beneficiaries and beneficiaries older than 85 years, which increases the validity of findings for those groups. MCBS has been conducted since 1991 by NORC (formerly the National Opinion Research Center) at the University of Chicago. Our sample consisted of all respondents aged 65 years or older with at least 1 hospitalization while enrolled. To simplify analyses, we used patients’ first hospitalization after MCBS enrollment. We used Medicare-linked MCBS data to identify hospitalization, which excluded participants with Medicare Advantage because linked claims were only available for participants with Medicare fee-for-service (FFS) plans. This study was considered exempt from review by the Mount Sinai institutional review board, which waived the requirement for obtaining informed consent, because all data were deidentified. We followed the Strengthening the Reporting of Observational Studies in Epidemiology (STROBE) reporting guideline.^32^

### Outcomes of Interest

To characterize the racial composition of hospitals, we linked the hospitals to the Centers for Medicare & Medicaid Services Medicare Inpatient Hospitals by Provider dataset from January 2011 through December 2020. The Medicare Inpatient Hospitals Public Use File summarizes the number of FFS beneficiaries who used services at a given hospital for 5 racial and ethnic groups (American Indian or Alaska Native, Asian or Pacific Islander, Black non-Hispanic, Hispanic, and White non-Hispanic) with an enhanced designation for race and ethnicity using the Research Triangle Institute algorithm.^11^ Our primary outcome of interest was hospitalization in a BSH vs a non-BSH commensurate with the uniquely high degree of residential, economic, and educational segregation for Black individuals in the US.^33^ However, for a more comprehensive picture of racial separation of care, we secondarily identified MSH, which disproportionately served patients from any racial and ethnic minority groups, including American Indian or Alaska Native, Asian, Black, Hispanic, or other. Thus, this second group also included BSH. Given the overlap between our 2 definitions, we present primary results on BSH with a summary of how results differ for MSH.

To determine whether a hospital qualified as a BSH or MSH, we used a comprehensive definition for our racial and ethnic groups of interest. For BSH, our racial and ethnic group of interest was non-Hispanic Black patients, whereas for MSH, our racial and ethnic group of interest was all patients from any racial or ethnic minority group. In keeping with prior literature, we included hospitals in the top decile of discharges of the race and ethnicity of interest.^12,34^ Furthermore, to capture hospitals that served a disproportion of patients of the racial and ethnic group of interest relative to the surrounding community but did not meet the national top decile threshold, we also included hospitals that discharged more than twice the surrounding HRR population percentage of that racial and ethnic group. Hospitals meeting this definition also have lower quality ratings.^35^

### Exposures of Interest

We defined neighborhood deprivation based on the patient’s zip code of residence, which was self-reported but verified by administrative claims and cross-walked to the Zip Code Tabulation Area (ZCTA). We used the social deprivation index (SDI [2015]), which is a multivariable index that quantifies neighborhood disadvantage.^36^ Higher SDI reflects greater disadvantage and is associated with worse health.^37^ Among the available measures, we used SDI because it was readily available at the ZCTA level. For interpretability, we categorized SDI into 5 groups (0-20, >20-40, >40-60, >60-80, and >80-100). We defined health care market residential segregation using the residential racial dissimilarity index (DI) of the HRR.^38,39^ There are approximately 300 HRRs in the US. We calculated the DI using data from the US Census Bureau American Community Survey aggregated to HRR. We categorized residential DI as low (<0.30), moderate (0.30-0.59), or high (≥0.60) segregation, consistent with prior work.^33^

### Covariates

We included patient race and ethnicity, age, sex, insurance, marital status, educational achievement, Elixhauser Comorbidity Index, and need for assistance with activities of daily living (ADLs). Race and ethnicity is an important confounder because it served as the basis for our definition of BSH and is associated with living in a racially segregated or disadvantaged area.^40^ Insurance is also a key covariate because insurance coverage influences where patients are hospitalized, and higher proportions of dually eligible Medicare and Medicaid patients are cared for in BSH.^11,41^ All patients in this analysis had Medicare FFS insurance but could vary in dual eligibility or having supplemental private insurance, both of which were thus included as covariates. We dichotomized marital status into currently married vs not, educational achievement into more than a high school diploma or equivalent vs not, and ADLs as needing assistance with none or at least 1 ADL. All variables were self-reported except the Elixhauser Comorbidity Index, which we calculated based on claims. At the health care market (HRR) level, we included the number and percentage of hospitals that were BSH. We also included US census region (Northeast, Midwest, South, or West), and whether the beneficiary lived in a major metropolitan area (defined as metropolitan area with a population of at least 50 000).

### Elective Hospitalizations

We defined an elective admission as one in which the claim was coded as elective. For confirmation, we included only admissions in which the patient did not have any claims from the emergency department as part of that admission.

### Statistical Analysis

First, we performed descriptive statistics identifying the percentage of respondents across patient, neighborhood, HRR, and regional characteristics who received care at a BSH. Next, we constructed bivariate logistic regression models testing each of our variables in association with receiving care in a BSH. Fully adjusted models included all aforementioned variables. To test effect modification, we included interaction terms in fully adjusted models for (1) race and ethnicity and SDI and (2) race and ethnicity and residential segregation. To test whether outcomes differed for elective vs nonelective admissions, we performed the same overall analyses in 2 models stratified by elective and nonelective.

We ran 2 sensitivity analyses: (1) a model excluding 2020 admissions given exceptionalism due to the COVID-19 pandemic and (2) models accounting for clustering at the HRR level. We did not use the latter model as our primary model because we aggregated 10 years of data for our analysis and clustering implies the region is static over that decade, which is belied by hundreds of hospital closures.^42^ Furthermore, the HRRs included in our analysis had a median of only 30 patients, ranging from 1 to 250, which may make results unreliable due to the influence of outliers.

For all analyses, we used a variance inflation factor of 2.0 as a cutoff for collinearity. We conducted our analyses from March 2023 through September 2025, using Stata, version 18.0 (StataCorp LLC). A 2-sided *P* < .05 was considered statistically significant.

## Results

### Characteristics of the Study Population

Our final sample included 8735 Medicare beneficiaries older than 65 years who were hospitalized from 2011 through 2020 (mean [SD] age, 79.6 [8.2] years; 4967 [56.9%] female, 3768 [43.1] male). A total of 756 (8.7%) were Black; 517 (5.9%), Hispanic; 7093 (81.2%), White; and 369 (4.2%), multiracial or other races (Table 1). In total, 1174 beneficiaries (13.4%) were hospitalized in BSH. Hospitalizations occurred in 1349 unique hospitals, 242 of which met our criteria for BSH. These hospitals served on average 27.8% Black patients, while non-BSH served on average 5.7% Black patients. At the patient level, 43.1% of hospitalizations of Black people and 9.8% of hospitalizations of White people occurred in BSH. We were missing SDI data for 195 patients (2.3%), and 78 of those patients were missing data for residential segregation. Fully adjusted models thus included 8540 patients. Patients without missing data were equally likely to be hospitalized at BSH and did not statistically differ by race or ethnicity.

**Table 1.** Description of Sample by Hospitalization Site

Characteristic	Patients, No. (%)		
	Total (N = 8735)	Non-BSH (n = 7561)	BSH (n = 1174)
Age, mean (SD), y	79.6 (8.2)	79.7 (8.2)	79.2 (8.3)
Sex			
Female	4967 (56.9)	4241 (56.1)	726 (61.8)
Male	3768 (43.1)	3320 (43.9)	448 (38.2)
Race and ethnicity			
Black	756 (8.7)	430 (5.7)	326 (27.8)
Hispanic	517 (5.9)	408 (5.4)	109 (9.3)
White	7093 (81.2)	6397 (84.6)	696 (59.3)
Multiracial or other^a^	369 (4.2)	326 (4.3)	43 (3.7)
Married currently	3878 (44.4)	3451 (45.6)	427 (36.4)
>High school diploma or equivalent	4173 (47.8)	3674 (48.6)	499 (42.5)
Medicaid	1538 (17.6)	1191 (15.8)	347 (29.6)
Private insurance	5119 (58.6)	4546 (60.1)	573 (48.8)
Elixhauser conditions, mean (SD), No.	8.7 (4.1)	8.7 (4.1)	8.9 (4.2)
Need assistance with ≥1 ADL	3020 (34.6)	2526 (33.4)	494 (42.1)
General health			
Excellent	807 (9.2)	715 (9.5)	92 (7.8)
Very good	2017 (3.1)	1796 (23.8)	221 (18.8)
Good	2572 (29.4)	2195 (29.0)	377 (32.1)
Fair	1686 (19.3)	1447 (19.1)	239 (20.4)
Poor	691 (7.9)	587 (7.8)	104 (8.9)
Living in metropolitan area	6517 (74.6)	5533 (73.2)	984 (83.8)
Social deprivation index^b^			
0-20 (Group 1)	1771 (21.0)	1632 (22.4)	139 (12.2)
>20-40 (Group 2)	1801 (21.4)	1630 (22.4)	171 (15.0)
>40-60 (Group 3)	1762 (20.9)	1574 (21.6)	188 (16.5)
>60-80 (Group 4)	1758 (20.9)	1469 (20.2)	289 (25.4)
>80-100 (Group 5)	1339 (15.9)	987 (13.5)	352 (30.9)
Residential DI, Black:White patients			
Low (<0.30)	796 (9.1)	700 (9.3)	96 (8.2)
Moderate (0.30-0.59)	6803 (77.9)	5979 (79.1)	824 (70.2)
High (≥0.60)	1058 (12.1)	814 (10.8)	244 (20.8)
US region			
Northeast	1652 (18.9)	1379 (18.2)	273 (23.3)
Midwest	2196 (25.1)	1972 (26.1)	224 (19.1)
South	3476 (39.8)	2882 (38.1)	594 (50.6)
West	1411 (16.2)	1328 (17.6)	83 (7.1)
Elective hospital admission	1929 (22.1)	1684 (22.3)	245 (20.9)

Abbreviations: ADL, activities of daily living; BSH, Black-serving hospital (hospital that disproportionately serves Black patients); DI, dissimilarity index.

^a^ Other included Alaska Native or American Indian, Asian, multiracial, and those who selected other.

^b^ Higher scores indicate greater deprivation.

Of 8735 beneficiaries, most (5119 [58.6%]) had supplemental private insurance and 1537 (17.6%) were dually eligible for Medicare and Medicaid. Respondents were nearly evenly distributed in terms of neighborhood disadvantage, with similar numbers in the 4 least disadvantaged groups and slightly fewer in the most disadvantaged group (20.9%-21.4% in neighborhoods with SDI scores of 0-20, >20-40, >40-60, and >60-80, and 15.9% in neighborhoods with SDI scores of >80-100). Patients primarily lived in HRRs with moderate residential segregation (6803 [77.9%] vs 1058 [12.1%] for high and 796 [9.1%] for low) (Table 1).

### Regression Models for BSH

In models fully adjusted for patient characteristics, HRR, and regional covariates, as neighborhood disadvantage and HRR residential segregation increased, so did the odds of receiving care in a BSH. The adjusted odds ratio [AOR] for most vs least disadvantaged neighborhoods were, 2.03 (95% CI, 1.56-2.63; *P* < .001), and the AOR for high vs low segregation HRR was 2.99 (95% CI, 1.98-4.52; *P* < .001) (Table 2).

**Table 2.** Factors Associated With Receiving Care in a BSH

Factor	OR (95% CI)	AOR (95% CI)^a^
Age	0.99 (0.99-1.00)	0.99 (0.98-1.00)
Female	1.27 (1.12-1.44)^b^	1.11 (0.95-1.30)
Race and ethnicity		
Black	6.97 (5.92-8.21)^b^	3.20 (2.59-3.96)^b^
Hispanic	2.46 (1.96-3.08)^b^	1.19 (0.89-1.61)
White	1 [Reference]	1 [Reference]
Multiracial or other^c^	1.21 (0.87-1.68)	0.85 (0.59-1.23)
Married currently		
No	1 [Reference]	1 [Reference]
Yes	0.68 (0.60-0.77)^b^	0.96 (0.81-1.13)
>High school diploma or equivalent		
No	1 [Reference]	1 [Reference]
Yes	0.78 (0.69-0.89)^b^	1.10 (0.94-1.30)
Medicaid	2.24 (1.95-2.58)^b^	1.28 (1.03-1.59)^d^
Private insurance	0.63 (0.56-0.72)^b^	0.93 (0.79-1.12)
No. of Elixhauser conditions	1.01 (1.00-1.03)	0.98 (0.96-1.00)^d^
Need assistance with ≥1 ADL		
No	1 [Reference]	1 [Reference]
Yes	1.44 (1.27-1.63)^b^	1.22 (1.03-1.44)^d^
Living in metropolitan area	1.90 (1.61-2.24)^b^	2.15 (1.75-2.64)^b^
Social deprivation index		
0-20 (Group 1)	1 [Reference]	1 [Reference]
>20-40 (Group 2)	1.23 (0.97-1.56)	1.11 (0.86-1.43)
>40-60 (Group 3)	1.40 (1.11-1.76)^b^	1.44 (1.12-1.85)^e^
>60-80 (Group 4)	2.31 (1.86-2.86)^b^	1.89 (1.48-2.41)^b^
>80-100 (Group 5)	4.19 (3,39-5.17)^b^	2.03 (1.56-2.63)^b^
Residential DI, Black:White patients		
Low (<0.30)	1 [Reference]	1 [Reference]
Moderate (0.30-0.59)	1.00 (0.80-1.26)	2.54 (1.81-3.58)^b^
High (≥0.60)	2.19 (1.69-2.83)^b^	2.92 (1.94-4.41)^b^
No. of BSH in HRR	1.11 (1.10-1.12)^b^	1.01 (0.99-1.04)
Percentage of BSH in HRR	1.03 (1.03-1.04)^b^	1.04 (1.04-1.04)^b^
US region		
Northeast	1 [Reference]	1 [Reference]
Midwest	0.57 (0.47-0.69)^b^	0.70 (0.55-0.89)^e^
South	1.04 (0.89-1.22)	0.73 (0.59-0.90)^e^
West	0.32 (0.24-0.41)^b^	0.63 (0.47-0.85)^b^

Abbreviations: ADL, activities of daily living; AOR, adjusted odds ratio; BSH, Black-serving hospital (hospital that disproportionately serves Black patients); DI, dissimilarity index; HRR, hospital referral region; OR, odds ratio.

^a^ Adjusted for all variables included in the table.

^b^ *P* ≤ .001.

^c^ Other included Alaska Native or American Indian, Asian, multiracial, and those who selected other.

^d^ *P* < .05.

^e^ *P* ≤ .01.

### Interaction Analyses

**Figure 1.**
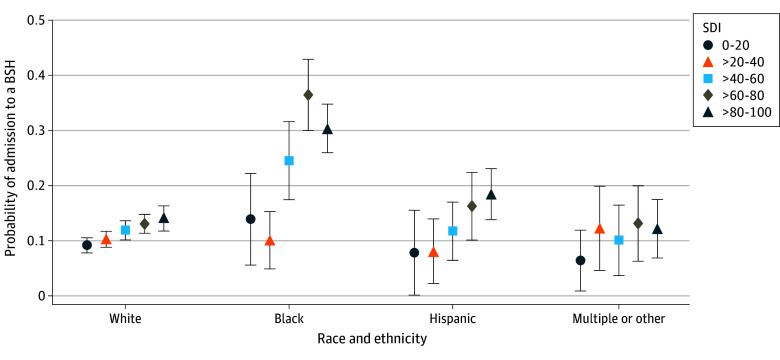
Dot-and-Whisker Plot of Interactions of Race and Ethnicity and Social Deprivation Index (SDI) for Black-Serving Hospitals (BSH) A BSH is a hospital that disproportionately serves Black patients. Higher SDI indicates greater disadvantage. Error bars represent 95% CIs.

**Figure 2.**
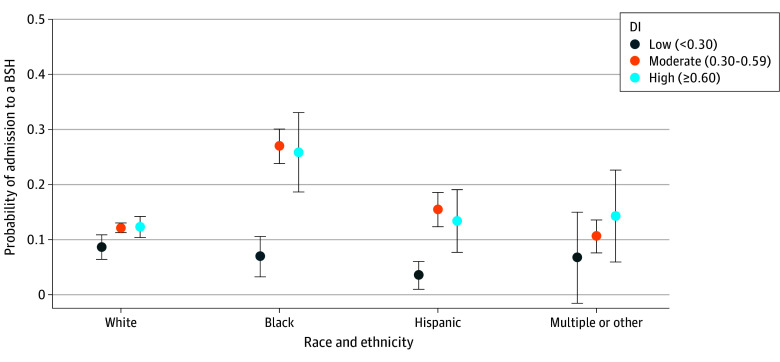
Dot-and-Whisker Plot of Interactions by Race and Ethnicity and Residential Dissimilarity Index (DI) for Black-Serving Hospitals (BSH) A BSH is a hospital that disproportionately serves Black patients. Error bars represent 95% CIs.

Race and ethnicity modified the association between neighborhood disadvantage and receiving care in a BSH (*P* = .04 for interaction) (Figure 1). In stratified models, in more disadvantaged neighborhoods compared with less disadvantaged neighborhoods, Black patients had higher odds of receiving care in a BSH (AOR, 2.77 [95% CI, 1.15-6.60]; *P* = .02); White patients also had higher odds, but it was lower than for Black patients (AOR, 1.70 [95% CI, 1.24-2.34]; *P* = .001). Race and ethnicity also modified the association between living in a racially segregated health care market and receiving care in a BSH (*P* = .001 for interaction) (Figure 2). In stratified models, in highly segregated HRRs compared with low segregation HRRs, Black patients had higher odds of receiving care in a BSH (AOR, 5.55 [95% CI, 1.86-16.57]; *P* = .002), whereas the AOR for White patients was 1.98 (95% CI, 1.23-3.18; *P* = .005).

### Elective Hospitalizations

In total, 1929 hospitalizations were elective, whereas 6806 were not. Using χ^2^ tests, we found that elective and nonelective hospitalizations were equally likely to be at BSH. However, we identified differences between elective and nonelective hospitalizations in the odds of an individual receiving care at BSH (eTable 1 in Supplement 1). For elective hospitalizations, neighborhood deprivation only had an association in group 5 in adjusted analyses and health care market residential segregation had no association, even in unadjusted models. Furthermore, race and ethnicity was not independently associated despite our definition of BSH based on the race and ethnicity of discharged patients. Results for nonelective hospitalizations closely mirrored our overall results. These 3-way interactions are illustrated in eFigures 1 and 2 in Supplement 1.

### Additional Findings in MSH

MSH had 1924 hospitalizations (22.0%). They served on average 37.4% racial and ethnic minority patients, whereas non-MSH served on average 13.5% racial and ethnic minority patients. Living in a moderate or highly racially segregated area (defined as a DI for White to racial and ethnic minority residents ≥0.30) was not independently associated with receiving care in an MSH. Otherwise, findings were largely the same as for BSH (Table 3 and eTable 2 in Supplement 1).

**Table 3.** Factors Associated With Receiving Care in an MSH

Factor	OR (95% CI)	AOR (95% CI)^a^
Age	1.00 (0.99-1.01)	1.00 (0.99-1.00)
Female sex	1.26 (1.13-1.39)^b^	1.15 (1.01-1.30)^c^
Race and ethnicity		
Black	4.66 (3.99-5.45)^b^	2.73 (2.27-3.29)^b^
Hispanic	5.10 (4.25-6.13)^b^	1.78 (1.40-2.25)^b^
White	1 [Reference]	1 [Reference]
Multiracial or other^d^	1.51 (1.18-1.93)^e^	0.90 (0.68-1.19)
Married currently		
No	1 [Reference]	1 [Reference]
Yes	0.72 (0.65-0.80)^b^	1.01 (0.88-1.15)
>High school diploma or equivalent		
No	1 [Reference]	1 [Reference]
Yes	0.78 (0.70-0.86)^b^	0.98 (0.86-1.11)
Medicaid	2.39 (2.12-2.69)^b^	1.24 (1.04-1.47)^c^
Private insurance	0.58 (0.53-0.64)^b^	0.90 (0.78-1.03)
No. of Elixhauser conditions	1.02 (1.01-1.03)^b^	0.98 (0.97-1.00)^c^
Need assistance with ≥1 ADL		
No	1 [Reference]	1 [Reference]
Yes	1.52 (1.37-1.69)^b^	1.21 (1.06-1.38)^e^
Living in metropolitan area	1.96 (1.72-2.24)^b^	1.67 (1.43-1.94)^b^
Social deprivation index		
0-20 (Group 1)	1 [Reference]	1 [Reference]
>20-40 (Group 2)	1.23 (1.01-1.48)^c^	1.04 (0.85-1.27)
>40-60 (Group 3)	1.74 (1.45-2.09)^b^	1.44 (1.18-1.76)^b^
>60-80 (Group 4)	2.42 (2.04-2.89)^b^	2.30 (1.89-2.79)^b^
>80-100 (Group 5)	4.74 (3.97-5.66)^b^	2.78 (2.26-3.41)^b^
Residential DI, racial and ethnic minority:White patients		
Low (DI <0.30)	1 [Reference]	1 [Reference]
Moderate or high (DI ≥0.30)	1.42 (1.24-1.62)^b^	0.99 (0.85-1.16)
No. of MSH in HRR	1.08 (1.07-1.09)^b^	0.99 (0.97-1.00)^c^
Percentage of MSH in HRR	1.03 (1.03-1.04)^b^	1.04 (1.03-1.04)^b^
US region		
Northeast	1 [Reference]	1 [Reference]
Midwest	0.70 (0.60-0.82)^b^	0.92 (0.76-1.12)
South	1.03 (0.90-1.19)	1.10 (0.93-1.31)
West	1.14 (0.97-1.35)	1.30 (1.06-1.60)

Abbreviations: ADL, activities of daily living; AOR, adjusted odds ratio; DI, dissimilarity index; HRR, hospital referral region; MSH, minority-serving hospital (hospital that disproportionately serves patients from any racial or ethnic minority group); OR, odds ratio.

^a^ Adjusted for all variables included in the table.

^b^ *P* ≤ .001.

^c^ *P* < .05.

^d^ Other included Alaska Native or American Indian, Asian, multiracial, and those who selected other.

^e^ *P* ≤ .01.

### Sensitivity Analyses

Excluding admissions in 2020 did not meaningfully change our findings except that neighborhood deprivation became nonsignificant for elective hospitalizations in BSH. Models accounting for clustering showed that HRR explained approximately one-third of the variation in whether patients were hospitalized in a Black-serving hospital (intraclass correlation coefficient, 0.34 [95% CI, 0.26-0.42]). In terms of the model outcomes, the primary difference between our original models and those accounting for clustering by HRR was that the latter no longer had an independent association between racial residential segregation and hospitalization in BSH (eTable 3 in Supplement 1). All interactions were consistent with our primary model, including that White people living in a highly or moderately segregated HRR were not more likely to be hospitalized in a BSH compared with those living in a low-segregation HRR, whereas Black people living in a moderately or highly segregated HRR were more likely to be hospitalized in a BSH.

## Discussion

In this cross-sectional study using 10 years of data from the MCBS, we found that neighborhood deprivation and health care market residential segregation were independently associated with receiving care in a BSH, but this association varied by patient race and ethnicity and by whether the hospitalization was elective or nonelective. These associations were also independent of patient characteristics, health care market, and regional covariates. For all admissions, the association between contextual factors and BSH admission differed by race. Specifically, Black patients living in the most disadvantaged neighborhoods had very high odds of receiving care in a BSH whereas White patients in the most disadvantaged neighborhoods had only moderately increased odds. Further, Black patients living in moderate and high segregation areas had higher odds of receiving care in BSH compared with those in low segregation areas, whereas White patients did not have any increased odds living in a high vs low segregation area. Intriguingly, elective admissions differed significantly from nonelective admissions. Indeed, race and ethnicity was not independently associated with receiving care in a BSH for elective admissions despite race and ethnicity of discharged patients being our means to define a hospital as BSH. Furthermore, living in a racially segregated area was not independently associated with elective hospitalization in a BSH nor was neighborhood deprivation (excluding 2020 admissions). These findings suggest that receipt of care in a BSH is one mechanism for racial health disparities resulting from living in a disadvantaged or segregated area.^5,43,44,45^ Our findings suggest that this is due to both differential exposure to these contextual factors and differential results of that exposure.^1^ Our work highlights the need for future research to understand why site of care differs for patients by race and ethnicity even if they live in similar areas and why contextual factors matter less for elective hospitalizations compared with nonelective hospitalizations. This may lead to interventions that decrease racial separation of care and ultimately decrease racial health disparities. In the meantime, policymakers should consider additional funding for BSH.

The differences between elective and nonelective hospitalizations suggest that racial sorting into different hospitals can be overcome when patients and health care professionals are able to make a considered choice.^26^ Mechanisms for this are unclear. Indeed, studies that have found racial differences in where patients choose to have elective surgeries or in physician referral patterns or recommendations for elective interventions based on patient race suggest that hospitalizations for elective procedures would have substantial racial sorting.^29,46,47^ More research is needed into why elective hospitalizations had less racial sorting to identify interventions that could also apply to nonelective hospitalizations.

For nonelective hospitalizations, neighborhood disadvantage and HRR residential segregation were independently associated with hospitalization in a BSH, although this differed by race. Research supporting mechanisms for racial sorting for nonelective hospitalizations may help explain these findings. Ambulances transport similarly located patients to different hospitals based on race and ethnicity, and there are racial differences in interfacility transfers regardless of patient characteristics.^48,49,50^ Thus, implicit bias may play a role.^51^ One study found that for protocolized conditions, racial differences in transfer to safety net hospitals were not present, suggesting a role of standardization for ambulance transport.^49^ However, this finding could be due to the modifiable areal unit problem.^52^ That is, it may be that Black and White patients live in different parts of racially segregated HRRs or disadvantaged neighborhoods, which are practically served by different hospitals.

### Limitations

This study has limitations. All respondents had FFS Medicare insurance, so we did not include patients with Medicare Advantage, thereby limiting our generalizability to FFS Medicare. However, during our sample period, most Medicare beneficiaries had FFS: from 75% in 2010 gradually decreasing to 58% in 2020.^53^ Including only patients with FFS allowed us to focus on upstream factors associated with receiving care at an MSH with less potential confounding from insurance. Future research could use all-payer data to provide insight into how insurance coverage could be leveraged to minimize racial sorting. This study is also limited by unmeasured confounders, including at the neighborhood level. Furthermore, while our measure of neighborhood deprivation (SDI) is a multivariable index and is associated with health outcomes, it does not include all variables that could influence use of BSH (eg, racialized economic inequality), which might explain our findings. Finally, further research is needed into hospitals that disproportionately serve American Indian and Alaska Native, Asian, or Hispanic patients, which may have different associated upstream factors. Our study found some evidence of this in that there was no independent association between living in a segregated HRR and receiving care at an MSH, underscoring the need for further research into hospitals disproportionately serving racial and ethnic minority groups other than Black individuals.

## Conclusions

In this cross-sectional study, neighborhood disadvantage and HRR residential racial segregation were key upstream factors associated with racial separation in care, but our findings suggest opportunities for intervention to reduce racial sorting. Further research is needed into why elective hospitalizations differed from nonelective hospitalizations and why living in a disadvantaged neighborhood or racially segregated hospital market was associated with higher odds of hospitalization at a BSH among Black people and lower odds or no association among White people. These racial differences in odds of BSH hospitalization for patients living in similar areas underscores that site of care may partially explain how place-based factors contribute to racial health disparities.^54,55^ Policymakers could also address neighborhood disadvantage and residential segregation. However, given the challenges inherent to ameliorating these factors,^28,33,56^ government investments in BSHs to support high quality of care may be warranted to improve racial health equity in the US.

## Notes

Supplement 1. **eTable 1.** Unadjusted and adjusted analyses for elective vs not elective for BSH **eTable 2.** Unadjusted and adjusted analyses for elective vs not elective for MSH **eFigure 1.** Interaction by race/ethnicity and SDI for elective vs not elective for BSH and MSH (4 panels) **eFigure 2.** Interaction by residential DI elective vs. not elective for BSH and MSH (4 panels) **eTable 3.** Factors associated with receiving care in a BSH: multilevel model clustering by HRR (random-intercept logistic model)

Supplement 2.
Data Sharing Statement
