# Supplementary material for: Upstream Factors Associated With Hospitalization in Black- and Minority-Serving Hospitals
Source: JAMA Netw Open. 2026 Jul 17;9(7):e2619544. doi: 10.1001/jamanetworkopen.2026.19544 (PMC13379745; doi:10.1001/jamanetworkopen.2026.19544)
Supplement: Supplement 1. — eTable 1. Unadjusted and adjusted analyses for elective vs not elective for BSH eTable 2. Unadjusted and adjusted analyses for elective vs not elective for MSH eFigure 1. Interaction by race/ethnicity and SDI for elective vs not elective for BSH and MSH (4 panels) eFigure 2. Interaction by residential DI elective vs. not elective for BSH and MSH (4 panels) eTable 3. Factors associated with receiving care in a BSH: multilevel model clustering by HRR (random-intercept logistic model) [file jamanetwopen-e2619544-s001.pdf]

## Supplementary Online Content

Holaday LW, Kung A, Chen Y, McKendrick K, Liu B, Siu A. Upstream factors associated with hospitalization in black- and minority-serving hospitals. *JAMA Netw Open*. 2026;9(7):e2619544. doi:10.1001/jamanetworkopen.2026.19544

**eTable 1.** Unadjusted and adjusted analyses for elective vs not elective for BSH
**eTable 2.** Unadjusted and adjusted analyses for elective vs not elective for MSH
**eFigure 1.** Interaction by race/ethnicity and SDI for elective vs not elective for BSH and MSH (4 panels)
**eFigure 2.** Interaction by residential DI elective vs. not elective for BSH and MSH (4 panels)
**eTable 3.** Factors associated with receiving care in a BSH: multilevel model clustering by HRR (random-intercept logistic model)

This supplementary material has been provided by the authors to give readers additional information about their work.

© 2026 Holaday LW et al. *JAMA Network Open*.

eTable 1. Unadjusted and adjusted analyses for elective vs not elective for BSH

| | Elective (n=1,929) | | Not Elective (n=6,806) | |
|---|---|---|---|---|
| | Unadjusted | Adjusted | Unadjusted | Adjusted |
| | Odds Ratio (95% CI) | Odds Ratio (95% CI) | Odds Ratio (95% CI) | Odds Ratio (95% CI) |
| Age | 1.00 (0.98, 1.02) | 0.99 (0.97, 1.02) | 0.99 (0.98, 1.00)[c] | 0.99 (0.98, 1.00) |
| Female | 1.29 (0.98, 1.69) | 1.12 (0.80, 1.56) | 1.26 (1.09, 1.45)[a] | 1.12 (0.93, 1.35) |
| Race (ref: White) | | | | |
| Black | 4.26 (2.75, 6.60)[a] | 1.64 (0.94, 2.86) | 7.71 (6.45, 9.22)[a] | 3.65 (2.89, 4.61)[a] |
| Hispanic | 2.01 (1.12, 3.62)[c] | 1.22 (0.56, 2.63) | 2.61 (2.04, 3.33)[a] | 1.21 (0.87, 1.68) |
| Multiple races/all other | 0.69 ( 0.27, 1.75) | 0.52 (0.18, 1.47) | 1.37 (0.96, 1.94) | 0.95 (0.64, 1.41) |
| Married currently (ref: no) | 0.73 (0.56, 0.95)[c] | 1.08 (0.76, 1.53) | 0.67 (0.58, 0.77)[a] | 0.91 (0.75, 1.10) |
| Greater than HS education (ref: no) | 1.11 ( 0.84, 1.48) | 1.40 (0.98, 2.00) | 0.72 (0.62, 0.83)[a] | 1.03 (0.86, 1.25) |
| Have any Medicaid | 2.59 (1.73, 3.88)[a] | 1.96 (1.08, 3.57)[c] | 2.22 (1.91, 2.58)[a] | 1.18 (0.94, 1.49) |
| Have any private insurance | 0.77 (0.58, 1.04) | 1.11 (0.74, 1.66) | 0.60 (0.52, 0.69)[a] | 0.89 (0.73, 1.09) |
| Number of Elixhauser conditions | 1.00 (0.97, 1.04) | 0.99 (0.95, 1.03) | 1.01 (1.00, 1.03) | 0.98 (0.96, 1.00) |
| Need assistance with 1+ ADL (ref: no) | 1.18 (0.86, 1.62) | 1.17 (0.80, 1.71) | 1.47 (1.28, 1.69)[a] | 1.27 (1.05, 1.53)[c] |
| Living in metropolitan area | 1.57 (1.13, 2.17)[b] | 1.84 (1.21, 2.79)[a] | 2.01 (1.67, 2.44)[a] | 2.27 (1.79, 2.87)[a] |
| SDI (ref: 0-20 group 1) | | | | |
| 20-40: group 2 | 1.03 (0.66, 1.58) | 1.00 (0.62, 1.61) | 1.36 (1.03, 1.80)[c] | 1.24 (0.91, 1.68) |
| 40-60: group 3 | 0.76 (0.48, 1.21) | 0.88 (0.52, 1.49) | 1.73 (1.32, 2.27)[a] | 1.80 (1.33, 2.44)[a] |
| 60-80: group 4 | 1.51 (1.02, 2.25)[c] | 1.41 (0.88, 2.25) | 2.74 (2.12, 3.45)[a] | 2.21 (1.64, 2.97)[a] |
| 80-100: group 5 | 2.67 (1.71, 4.15)[a] | 1.84 (1.06, 3.21)[c] | 4.92 (3.83, 6.32)[a] | 2.23 (1.65, 3.02)[a] |
| Residential DI Black/White (ref: low) | | | | |
| Moderate | 0.73 (0.46, 1.16) | 1.50 (0.77, 2.94) | 1.10 (0.85, 1.42) | 3.01 (2.07, 4.65)[a] |
| High | 0.87 (0.49, 1.55) | 1.02 (0.42, 2.48) | 2.71 (2.03, 3.63)[a] | 3.95 (2.45, 6.36)[a] |
| Number of BSH in HRR | 1.13 (1.10, 1.16)[a] | 1.02 (0.97, 1.06) | 1.12 (1.11, 1.13)[a] | 1.01 (0.99, 1.04) |
| Percent of BSH in HRR | 1.04 (1.03, 1.04)[a] | 1.04 (1.03, 1.04)[a] | 1.04 (1.04, 1.04)[a] | 1.04 (1.04, 1.05)[a] |
| Region (ref: Northeast) | | | | |
| Midwest | 0.38 ( 0.24, 0.60)[a] | 0.68 (0.39, 1.17) | 0.64 (0.52, 0.79)[a] | 0.69 (0.52, 0.91)[b] |
| South | 1.03 ( 0.71, 1.49) | 0.86 (0.54, 1.37) | 1.04 (0.87, 1.24) | 0.68 (0.53, 0.87)[b] |
| West | 0.33 (0.19, 0.56)[a] | 0.56 (0.30, 1.05) | 0.31 (0.23, 0.42)[a] | 0.66 (0.47, 0.93)[c] |

a. $P \leq 0.001$
b. $P \leq 0.01$
c. $P < 0.05$

© 2026 Holaday LW et al. *JAMA Network Open*.

eTable 2. Unadjusted and adjusted analyses for elective vs not elective for MSH

| | Elective (n=1,929) | | Not Elective (n=6,806) | |
|---|---|---|---|---|
| | Unadjusted | Adjusted | Unadjusted | Adjusted |
| | Odds Ratio (95% CI) | Odds Ratio (95% CI) | Odds Ratio (95% CI) | Odds Ratio (95% CI) |
| Age | 1.01 (1.00, 1.03) | 1.01 (0.99, 1.03) | 1.00 (0.99, 1.00) | 0.99 (0.99, 1.00) |
| Female | 1.29 (1.02, 1.62)[c] | 1.13 (0.86, 1.48) | 1.24 (1.10, 1.39)[a] | 1.16 (1.01, 1.34)[c] |
| Race (ref: White) | | | | |
| Black | 3.11 (2.04, 4.73)[a] | 1.63 (0.98, 2.71) | 4.96 (4.19, 5.87)[a] | 2.95 (2.41, 3.61)[a] |
| Hispanic | 3.50 (2.17, 5.65)[a] | 1.57 (0.86, 2.84) | 5.44 (4.45, 6.64)[a] | 1.79 (1.38, 2.32)[a] |
| Multiple race/all other | 1.17 (0.61, 2.22) | 0.83 (0.41, 1.71) | 1.58 (1.21, 2.08)[a] | 0.91 (0.67, 1.24) |
| Married currently (ref: no) | 0.75 (0.60, 0.94)[c] | 1.02 (0.77, 1.36) | 0.73 (0.65, 0.82)[a] | 0.99 (0.85, 1.16) |
| Greater than HS education (ref: no) | 1.10 (0.87, 1.40) | 1.16 (0.87, 1.54) | 0.73 (0.65, 0.82)[a] | 0.93 (0.80, 1.08) |
| Have any Medicaid | 2.34 (1.62, 3.38)[a] | 1.87 (1.14, 3.07)[c] | 2.36 (2.07, 2.68)[a] | 1.15 (0.96, 1.39) |
| Have any private insurance | 0.67 (0.53, 0.86)[a] | 0.85 (0.62, 1.15) | 0.57 (0.51, 0.64)[a] | 0.92 (0.79, 1.07) |
| Number of Elixhauser conditions | 1.00 (0.98, 1.03) | 0.98 (0.95, 1.02) | 1.02 (1.00, 1.03)[c] | 0.98 (0.97, 1.00) |
| Need assistance with 1+ ADL (ref: no) | 1.23 (0.94, 1.60) | 1.02 (0.75, 1.39) | 1.55 (1.38, 1.73)[a] | 1.26 (1.09, 1.46)[a] |
| Living in metropolitan area | 1.58 (1.21, 2.07)[a] | 1.46 (1.07, 2.00)[c] | 2.08 (1.78, 2.41)[a] | 1.75 (1.47, 2.08)[a] |
| SDI group (ref: 0-20 group 1) | | | | |
| 20-40: group 2 | 1.18 (0.82, 1.70) | 1.05 (0.71, 1.55) | 1.26 (1.01, 1.57)[c] | 1.08 (0.85, 1.37) |
| 40-60: group 3 | 1.17 (0.81, 1.69) | 1.06 (0.70, 1.59) | 1.97 (1.60, 2.44)[a] | 1.63 (1.29, 2.05)[a] |
| 60-80: group 4 | 1.55 (1.10, 2.19)[c] | 1.55 (1.05, 2.29) | 2.81 (2.29, 3.45)[a] | 2.65 (2.11, 3.32)[a] |
| 80-100: group 5 | 2.90 (1.96, 4.29)[a] | 2.49 (1.58, 3.92)[a] | 5.34 (4.36, 6.55)[a] | 3.00 (2.37, 3.79)[a] |
| Residential DI Non-White/White (ref: low) | | | | |
| Moderate/High | 1.75 (1.30, 2.36)[a] | 1.32 (0.94, 1.86) | 1.33 (1.15, 1.54)[a] | 0.91 (0.76, 1.08) |
| Number of MSH in HRR | 1.07 (1.05, 1.08)[a] | 0.96 (0.93, 0.99)[b] | 1.08 (1.07, 1.09)[a] | 0.99 (0.98, 1.01) |
| Percent of MSH in HRR | 1.03 (1.03, 1.04)[a] | 1.04 (1.03, 1.05)[a] | 1.03 (1.03, 1.04)[a] | 1.04 (1.03, 1.04)[a] |
| Region (ref: Northeast) | | | | |
| Midwest | 0.59 (0.40, 0.88)[b] | 0.86 (0.55, 1.36) | 0.75 (0.63, 0.89)[a] | 0.93 (0.76, 1.15) |
| South | 1.14 (0.81, 1.60) | 1.50 (0.99, 2.28) | 1.02 (0.88, 1.19) | 1.01 (0.84, 1.23) |
| West | 1.08 (0.72, 1.60) | 1.52 (0.95, 2.44) | 1.18 (0.98, 1.42) | 1.25 (0.99 1.58) |

a. $P \leq 0.001$
b. $P \leq 0.01$
c. $P < 0.05$

© 2026 Holaday LW et al. *JAMA Network Open*.

eFigure 1. Interaction by race/ethnicity and SDI for elective vs not elective for BSH and MSH (4 panels)

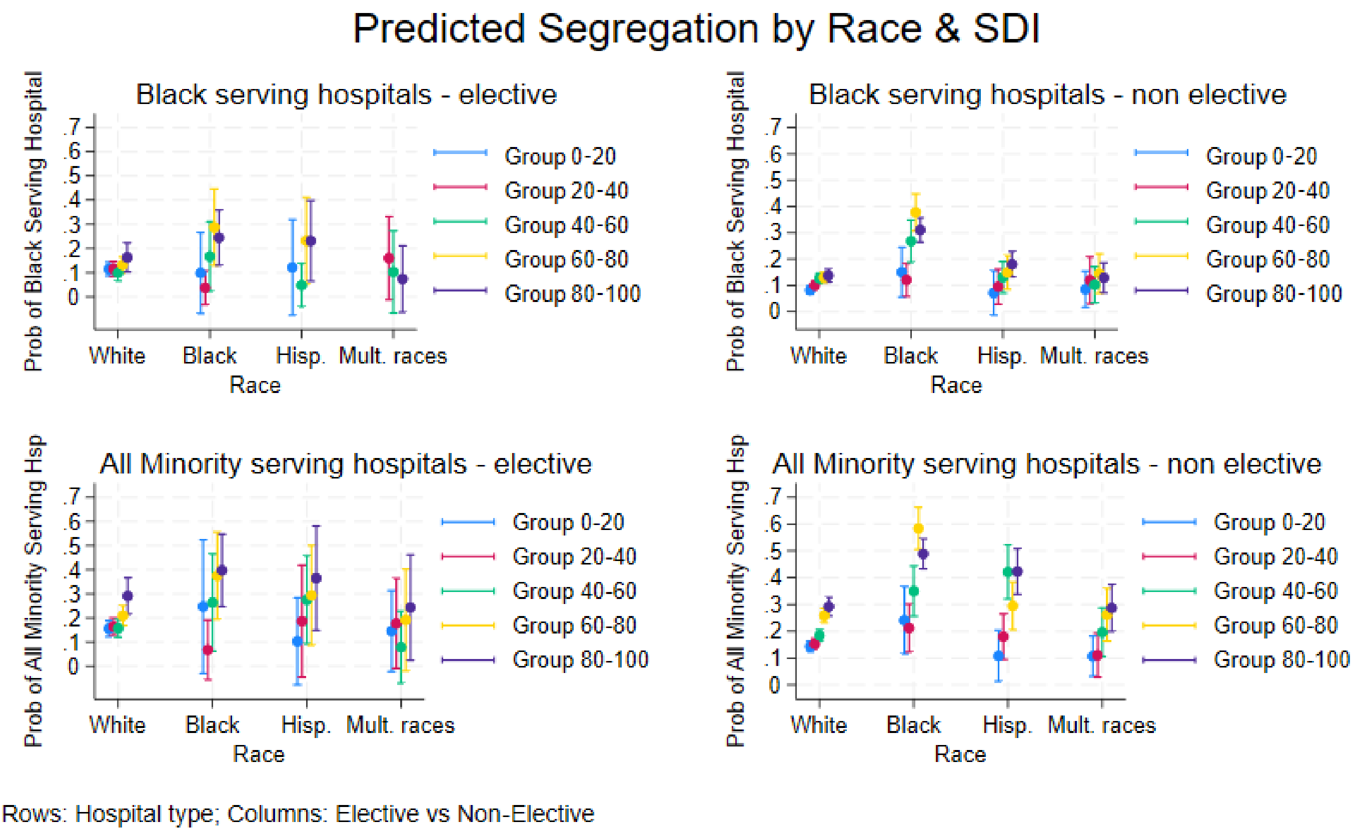


© 2026 Holaday LW et al. *JAMA Network Open*.

eFigure 2. Interaction by residential DI elective vs. not elective for BSH and MSH (4 panels)

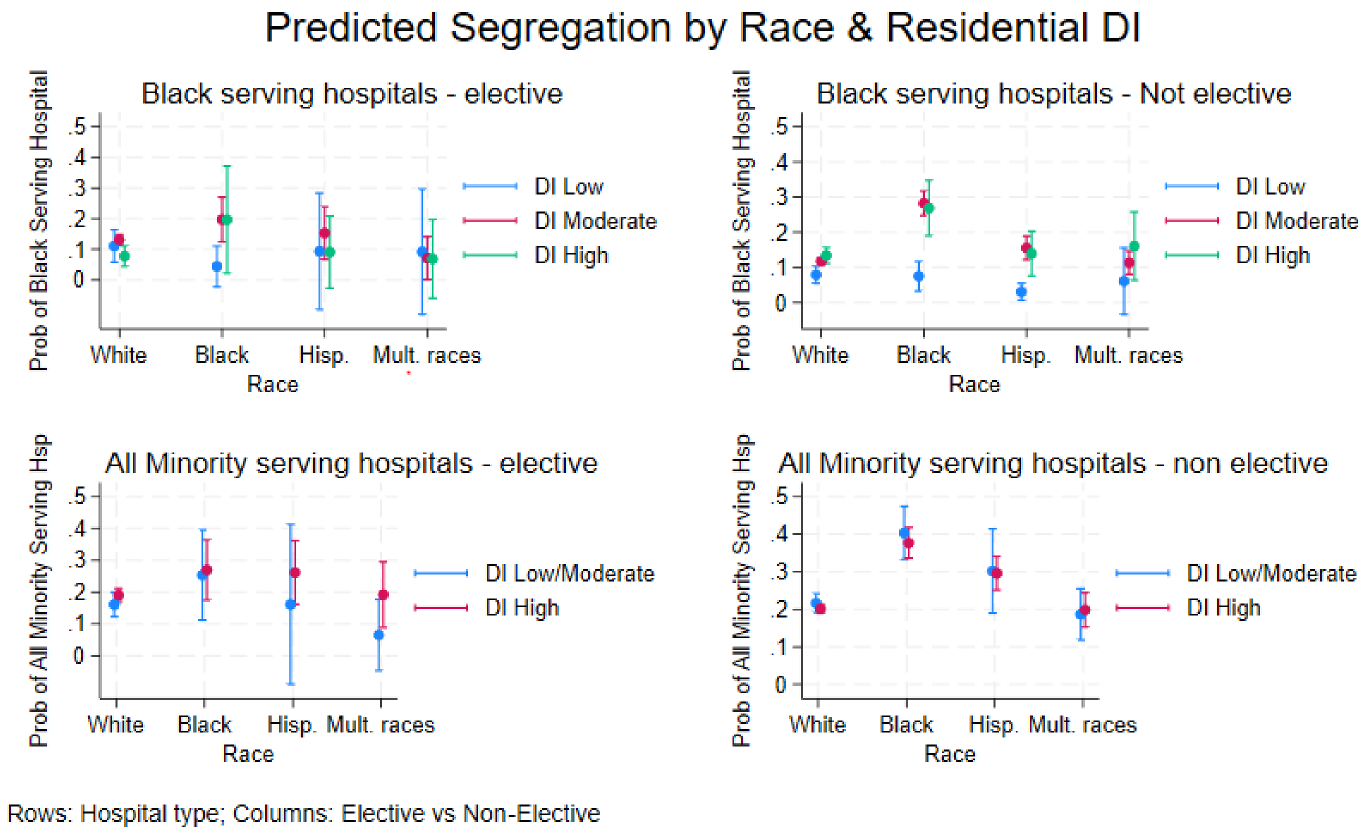


© 2026 Holaday LW et al. *JAMA Network Open*.

eTable 3. Factors associated with receiving care in a BSH: Multilevel model clustering by HRR (random-intercept logistic model)

| | Adjusted |
|---|---|
| | Odds Ratio (95% CI) |
| Age | 0.99 (0.98, 1.00) |
| Female | 1.14 (0.96, 1.35) |
| Race (ref: White) | |
| Black | 3.87 (3.03, 4.98)[a] |
| Hispanic | 1.30 (0.93, 1.80) |
| Multiple races/all other | 1.03 (0.70, 1.53) |
| Married currently (ref: no) | 1.05 (0.88, 1.27) |
| Greater than HS education (ref: no) | 0.79 (0.53, 1.18) |
| Have any Medicaid | 1.16 (0.92, 1.47) |
| Have any private insurance | 0.90 (0.74, 1.09) |
| Number of Elixhauser conditions | 0.98 (0.96, 1.00) |
| Need assistance with 1+ ADL (ref: no) | 1.28 (1.07, 1.54)[b] |
| Living in metropolitan area | 1.74 (1.23, 2.46)[b] |
| SDI (ref: 0-20 group 1) | |
| 20-40: group 2 | 1.27 (0.96, 1.70) |
| 40-60: group 3 | 1.70 (1.26, 2.27)[a] |
| 60-80: group 4 | 1.76 (1.31, 2.36)[a] |
| 80-100: group 5 | 2.79 (2.05, 3.80)[a] |
| Residential DI Black/White (ref: low) | |
| Moderate | 1.63 (0.73, 3.62) |
| High | 2.89 (0.90, 9.26) |
| Number of BSH in HRR | 1.04 (0.95, 1.14) |
| Percent of BSH in HRR | 1.04 (1.03, 1.06)[a] |
| Region (ref: Northeast) | |
| Midwest | 0.56 (0.28, 1.15) |
| South | 0.86 (0.45, 1.66) |
| West | 0.76 (0.33, 1.75) |

a. $P \leq 0.001$
b. $P \leq 0.01$
c. $P < 0.05$

© 2026 Holaday LW et al. *JAMA Network Open*.
