# Supplementary material for: Upstream Factors Associated With Hospitalization in Black- and Minority-Serving Hospitals
Source: JAMA Netw Open. 2026 Jul 17;9(7):e2619544. doi: 10.1001/jamanetworkopen.2026.19544 (PMC13379745; doi:10.1001/jamanetworkopen.2026.19544)
Supplement: Supplement 2. — Data Sharing Statement [file jamanetwopen-e2619544-s002.pdf]

Data Sharing Statement

Holaday. Upstream Factors Associated With Hospitalization in Black- and Minority-Serving Hospitals. *JAMA Netw Open*. Published July 17, 2026.
doi:10.1001/jamanetworkopen.2026.19544

Data
Data available: No

Additional Information
Explanation for why data not available: Data is available from CMS through a DUA
